# Inflammatory indicators derived from complete blood counts in relation to osteoarthritis prevalence: findings from the NHANES 2007–2020 cross-sectional survey

**DOI:** 10.3389/ebm.2025.10815

**Published:** 2026-01-13

**Authors:** Zimo Ye, Tianran Zhao, Xinlin Huang, Yingxue Song, Luyi Cheng, Yunyi Liu, Mingde Qiu, Ruke Long, Weihao Chen, Yu Wang, Hao Xie, Lei Fan, Xiaolong Hu

**Affiliations:** 1 Huiqiao Medical Center, Nanfang Hospital, Southern Medical University, Guangzhou, Guangdong, China; 2 The Second School of Clinical Medicine, Southern Medical University, Guangzhou, Guangdong, China; 3 The School of Public Health, Southern Medical University, Guangzhou, Guangdong, China; 4 Department of Orthopedics, Affiliated Hospital of Jilin Medical College, Jilin City, Jilin Province, China; 5 Department of Orthopedic Surgery, Nanfang Hospital, Southern Medical University, Guangzhou, Guangdong, China

**Keywords:** epidemiology, inflammation, leukocyte, orthopaedics, osteoarthritis

## Abstract

Although multiple studies have confirmed the importance of chronic low-grade inflammation in the development of osteoarthritis (OA), the association between complete blood count (CBC)-derived inflammatory indicators and osteoarthritis prevalence remains unclear. The present study aims to explore the association between CBC-derived inflammatory indicators and OA prevalence. We used NHANES data from 2007 to 2020 for a cross-sectional analysis. Multivariate logistic regression models were used to evaluate the association between CBC-derived inflammatory indicators and OA prevalence. Restricted cubic spline function (RCS) and threshold analysis were used to assess potential nonlinear associations. In addition, subgroup and sensitivity analyses were performed to assess the stability of the results. Finally, we used LASSO regression to identify the variables most associated with OA outcomes to construct a prediction model, and the model’s validity was verified. Among the 24,112 patients in this study, 3,195 were diagnosed with OA. In the adjusted model, multivariate logistic regression analysis showed that 5 inflammatory indicators (SII, SIRI, MLR, NMLR, NLR) were positively associated with OA prevalence. RCS and threshold analysis showed nonlinear associations between (SII, NMLR, NLR) and OA prevalence. After variable screening, we established an OA risk prediction model with an area under the curve (AUC) of 0.735 (95% CI: 0.726–0.744). Both the decision and calibration curve showed that the model had good clinical significance. The Present study suggests that CBC-derived inflammatory indicators are statistically associated with OA prevalence. Furthermore, MLR and NMLR could be valuable predictors of OA and offer novel perspectives on its assessment and treatment.

## Impact statement

This study challenges the traditional view of osteoarthritis as merely a consequence of joint wear by demonstrating significant associations between systemic inflammation (measured through widely available CBC indicators) and OA prevalence in a major U.S. cohort. It identifies novel nonlinear associations of SII, NMLR, and NLR with OA prevalence, suggesting complex dynamic relationships between cellular inflammation and OA that merit further mechanistic investigation. By integrating MLR and NMLR into a clinically applicable prediction model with strong discriminatory power (AUC = 0.735), this work provides an accessible tool for early OA risk assessment using routine blood parameters. These findings underscore the role of immune-inflammatory processes in OA and may inform future strategies for prevention, early intervention, and mechanistic research.

## Introduction

Osteoarthritis (OA) is a common chronic joint disease characterized by pathological changes in articular cartilage, subchondral bone remodeling, and synovial abnormalities, which eventually lead to clinically significant pain and functional impairment [1]. According to Hunter et al, more than 500 million people are affected by OA worldwide [2]. A recent study by Sun et al. [3] found that nearly 14 million people in the United States suffer from knee OA, which has led to a heavy public health burden. Currently, drug therapy is the main treatment for early and middle-stage OA, while joint replacement surgery remains the only effective treatment for end-stage disease [4]. However, postoperative joint function may be unsatisfactory, and patients face the problem of limited lifespan of the replacement joint, which, in addition to pain, leads to reoperation and increased costs. Therefore, identifying modifiable factors is crucial to developing feasible strategies to delay the progression of OA and reduce the associated social and economic burden and negative impact on patients [5]. Currently, there is no recognized risk predictor for OA. To address this gap, we conducted the present study to examine whether CBC-derived inflammatory indicators are associated with OA risk and to identify predictors that may be clinically useful. In addition, the findings may provide useful perspectives and references for future mechanistic research.

OA is a multifactorial disease influenced by aging, genetic susceptibility, obesity, and, in particular, inflammatory processes [6, 7], with specific inflammatory biomarkers being key indicators of disease progression. Emerging evidence indicates that TNF-α and IL-1β are key inflammatory mediators in OA. These cytokines interact with multiple signaling pathways to promote further cytokine release and are involved in the pathogenesis of OA [8]. Systemic inflammatory responses can be assessed using complete blood count (CBC)-derived inflammatory indicators, which are calculated from routine blood count parameters and include systemic immune inflammatory index (SII), systemic inflammatory response index (SIRI), monocyte-to-lymphocyte ratio (MLR), (neutrophil + monocyte)-to-lymphocyte ratio (NMLR), neutrophil-to-lymphocyte ratio (NLR), and derived NLR (dNLR). Peripheral platelets, lymphocytes, and neutrophils together comprise these inflammatory markers, which constitute a comprehensive set of parameters used for prognosis. Compared with a single inflammatory signal, they can more comprehensively reflect the host’s immune-inflammatory status [9, 10], highlighting their potential as key indicators of the body’s inflammatory and immune status. In addition, since CBC-derived inflammatory indicators are readily obtained from routine complete blood counts, they are easy to use in clinical applications.

It is noteworthy that multiple complete blood count (CBC)-derived inflammatory indicators are crucial for diagnosing and treating various diseases [11–13]. A study by Ke et al. [14] found that elevated NLR and SII levels in asthma patients were associated with an increased likelihood of respiratory disease-related death. In addition, studies have found that NLR and PLR are closely associated with PASI scores in patients with psoriasis, suggesting that they can reflect systemic inflammation [15]. Previous studies on OA have mainly focused on the association between individual inflammatory indicators [16–18] and disease status. However, this approach focuses on a limited date range and specific indices [19], which may lead to false findings. Therefore, we comprehensively investigated the association between CBC-derived inflammatory indicators and OA prevalence and tested their predictive performance. We hypothesized that the OA patients have a higher level of CBC-derived inflammatory indicators.

## Materials and methods

### Study design and population

The present study used publicly available datasets obtained from the NHANES^1^ platform. All procedures were approved by the institutional review board of the National Center for Health Statistics, and participants were requested to submit informed consent [20].

**FIGURE 1:**
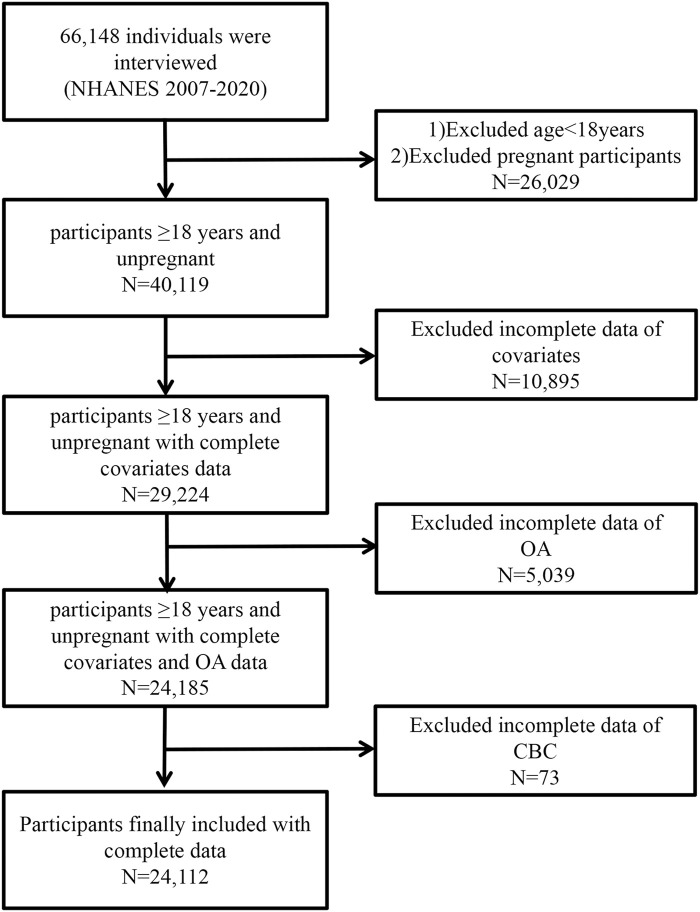
Flow diagram of the selection of eligible participants.

Of the NHANES 2007–2020 data, 66,148 individuals were recognized. The exclusion criteria were as follows: (1) individuals younger than 18 years or pregnant; (n = 26,069); (2) participants with missing covariate data (n = 10,895); (3) participants without osteoarthritis information (n = 5,039); (4) participants without CBC data (n = 73). In the end, 24,112 individuals participated in the present research. Figure 1 depicts the complete data selection procedure. Every piece of statistical data used in the present study is publicly accessible and demographically weighted for further study.

### Definition of CBC-derived inflammatory indicators

The NHANES mobile examination center (MEC) used the Beckman Coulter DxH 800 device to analyze CBCs on blood samples and report blood cell distribution for each individual. The procedure was overseen by qualified medical staff. Subsequently, using certain mathematical formulae based on the complete counts of various blood cell subpopulations, we computed the following inflammatory indicators: SII, SIRI, MLR, NMLR, NLR, and dNLR. SII = neutrophil counts × platelet counts/lymphocyte counts, SIRI = neutrophil counts × monocyte counts/lymphocyte counts, MLR = monocyte counts/lymphocyte counts, NMLR = (neutrophil counts + monocyte counts)/lymphocyte counts, NLR = neutrophil counts/lymphocyte counts, dNLR = neutrophil counts/(white blood cell counts-lymphocyte counts).

### Definition of osteoarthritis

A questionnaire survey was used to determine whether a subject had OA. “Did a doctor or other healthcare professional ever tell you that you have arthritis?” is the first question that participants would be asked. The question of “What type of arthritis was it?” would be required if the response to the first question was “yes.” Each individual was assigned to an OA or non-OA subgroup based on their responses to the two questions. The individuals were placed in the non-OA subgroup if they answered “no” to the first question. The OA group was assigned to the participant if they answered “yes” to the first question and “OA” to the second. In comparison, participants were placed in the non-OA group if they answered “no” to the first question.

### Definition of covariates

Covariates for statistical analysis included demographic statistics (age, gender, race, marital status, educational level), life behavior traits (energy intake, smoking status, and drinking status), body mass index (BMI), concurrent illnesses (diabetes and hypertension), and laboratory indicators that could influence the results (albumin, urine acid, and urine creatinine). From the demographic statistics, the age of each participant was gathered. The individuals were then further divided into two subgroups: Age <60 and 60 ≤ Age [21]. Every individual involved in the present research was older than 18. The participants were stratified by race into five subgroups: non-Hispanic White, non-Hispanic Black, other Hispanic, Mexican American, and other race. Marital status was categorized into three states: never married, married/living with partner, and widowed/divorced/separated. Education level was further classified into five subgroups: less than 9th grade, 9–11th grade, high school graduate/GED or equivalent, some college or AA degree, and college graduate or above. The initial 24-h recall questionnaire enabled the collection of data on energy intake. Using a smoking questionnaire, participants’ smoking status was categorized as never smoking (no more than 100 tobacco products), previous smoking (greater than 100 tobacco products, not smoking at the moment), and present smoking (greater than 100 tobacco products, currently smoking). Each participant’s BMI, calculated as weight divided by height squared (kg/m2), was derived from the examination statistics. According to the drinking questionnaire, the drinking status was classified as no drinking (less than one time a week), mild (one to three times a week), and intense (more than three times a week) [22]. Diagnosis of diabetes was based on antidiabetic medication use, questionnaire results, or a fasting plasma glucose level >7.0 mmol/L. Once systolic pressure was greater than 140 mmHg or diastolic pressure was greater than 90 mmHg, the diagnosis of hypertension was established. The NHANES program’s laboratory test data included statistics on albumin, creatinine, and calcium.

### Statistical analysis

Weighted analyses were conducted in accordance with NHANES guidelines due to the complex sample survey. To examine baseline differences between normal and OA groups, a weighted chi-square test (categoric variables) and the weighted Wilcoxon Rank Sum Test (continuous variables) were employed. The association between CBC-derived inflammatory indicators and the OA prevalence was examined using weighted multivariate logistic regression: no variables were adjusted for in the crude model. Model 1 was adjusted for age, gender, race, marital status, and education level; Model 2 was adjusted for age, gender, race, marital status, education level, smoking status, drinking status, energy intake, BMI, diabetes, and hypertension; and Model 3 was adjusted for age, gender, race, marital status, education level, smoking status, drinking status, energy intake, BMI, diabetes, hypertension, albumin, creatinine, and calcium. CBC-derived inflammatory indicators were considered both continuous and categorical (trichotomies). To assess whether the logistic regression model was affected by multicollinearity, we examined pairwise correlations among the continuous variables included in the fully adjusted model by constructing correlation matrices (Supplementary Figure S1). In addition, we calculated the variance inflation factor (VIF) for all variables in the fully adjusted model, as shown in Supplementary Table S1.

To explore nonlinear associations, our study also uses restricted cubic splines (RCS). We evaluated spline specifications with 3, 4, and 5 knots and selected the 3-knot structure based on the lowest Akaike Information Criterion (AIC) together with model parsimony. The AIC values for the fully adjusted models under the 3-, 4-, and 5-knot specifications are provided in Supplementary Table S2. The log-likelihood ratio test was used to compare two-segment linear regression models (segmented regression models) with a single linear model (nonsegmented model) for indicators with nonlinear relationships. Threshold effects were then computed. The association between CBC-derived inflammatory indicators and OA across subgroups was subsequently investigated using subgroup analysis. Age, gender, race, marital status, education level, diabetes, and hypertension were among the stratification variables. Then, sensitivity analyses were conducted to evaluate the reliability of our studies. To reconfirm the correlation between CBC inflammatory indicators and OA prevalence and eliminate the influence of confounding factors, we performed multiple imputation for missing data using the mice package in R. We applied the default settings of the defaultMethod parameter, which imputes continuous variables, binary variables, unordered categorical variables, and ordered categorical variables using the package’s corresponding default methods. Density plots comparing the distributions of observed and imputed values are provided in Supplementary Figure S2.

Subsequently, a predictive model was constructed and validated. First, variables were screened and regressed using LASSO regression, and the optimal lambda was selected via cross-validation to determine the best prediction model. The risk prediction model was visually represented as a nomogram that quantifies the overall likelihood of developing OA by assigning weighted scores to various factors based on their risk contributions. To construct the nomogram, the VIFs for each predictor (Supplementary Table S3) were also computed to assess potential multicollinearity further. Finally, the specificity and sensitivity of the model were evaluated by ROC curve analysis and the corresponding AUC. Internal validation was also performed using the bootstrap resampling method, with 1,000 bootstrap samples generated to estimate the model’s accuracy and stability. In addition, decision curve analysis and calibration curves were performed to evaluate the model’s clinical utility and the accuracy of its predicted probability.

All analyses were conducted using R software (version 4.4.2), and a two-sided P value <0.05 was considered statistically significant. Moreover, we completed this checklist (Supplementary Material) in accordance with the STROBE statement to ensure completeness of reporting of our cross-sectional study.

## Results

### Baseline characteristics of the study population

The present data set included 24,112 participants (23.8% were older than 60 years, and 50.2% were male), of whom 3,195 had OA, yielding a weighted prevalence of 14.28%. The median values of CBC-derived inflammatory indicators [Q1, Q3] were calculated as follows: SII 462.68 [336.54, 640.64], SIRI 1.04 [0.73, 1.50], MLR 0.26 [0.21, 0.33], NMLR 2.22 [1.73, 2.87], NLR 1.95 [1.50, 2.55], dNLR 0.84 [0.80, 0.87]. CBC-derived inflammatory indicators (except dNLR) were significantly increased in OA patients compared with non-OA patients (p < 0.001). The body mass index (BMI) and age of OA patients were considerably greater than those of the non-OA group. Additionally, there were substantially more women in the OA group than in the non-OA group. Baseline characteristics such as race, marital status, education level, smoking and drinking status, diabetes, hypertension, energy intake, and albumin showed significant statistical differences among different OA statuses (Table 1).

**TABLE 1:** Baseline characteristics of participants according to osteoarthritis.

Characteristic	Overall	Osteoarthritis		p-value^a^
		No	Yes	
Unweighted number	24,122	20,927	3,195	​
Weighted number	161,493,424	138,427,174	23,066,250	​
Demographic data				
Age,mean (SD)	46.08 (16.77)	43.44 (15.87)	61.92 (12.78)	<0.001
Age,%	​	​	​	<0.001
<60 years	76.2	82.4	38.9	​
>60 years	23.8	17.6	61.1	​
Gender,%	​	​	​	<0.001
Male	50.2	52.8	34.7	​
Female	49.8	47.2	65.3	​
Race,%	​	​	​	<0.001
Mexican American	8.8	9.8	2.6	​
Non-hispanic white	67.6	64.9	83.3	​
Non-hispanic black	9.9	10.6	6.0	​
Other hispanic	6.0	6.6	2.8	​
Other race	7.7	8.1	5.2	​
Educational level,%				
Less than 9th grade	4.2	4.3	3.2	0.063
9–11th grade	9.4	9.5	8.3	​
High school graduate/GED or equivalent	23	22.9	23.6	​
Some college or AA degree	31.3	31.0	33.2	​
College graduate or above	32.2	32.3	31.6	​
Marital status,%	​	​	​	<0.001
Married/Living with partner	63.5	63.3	65.3	​
Widowed/Divorced/Separated	16.6	14.6	28.2	​
Never married	19.9	22.1	6.5	​
Concurrent disease				
Diabetes,%	​	​	​	<0.001
Yes	10.4	8.9	19.6	​
No	89.6	91.1	80.4	​
Hypertension,%	​	​	​	<0.001
Yes	32.4	28.0	59.2	​
No	67.6	72.0	40.8	​
Life behavior characteristics				
BMI, median [IQR]	27.77 [24.13, 32.30]	27.50 [23.96, 31.90]	29.49 [25.60, 34.10]	<0.001
Energy intake, median [IQR]	2034.00 [1,521.00, 2,692.00]	2061.00 [1,546.00, 2,730.04]	1854.72 [1,387.91, 2,419.98]	<0.001
Smoking status,%	​	​	​	<0.001
Never smoking	57.2	58.8	47.4	​
Previous smoking	24.2	22.1	36.8	​
Present smoking	18.7	19.2	15.8	​
Drinking status,%	​	​	​	0.449
No	89.5	89.5	89.3	​
Mild	5.9	6.0	89.3	​
Intense	4.6	4.5	5.2	​
Laboratory data				
Uric acid, median [IQR]	5.30 [4.40, 6.30]	5.30 [4.40, 6.30]	5.30 [4.40, 6.20]	0.842
Albumin, median [IQR]	43.00 [41.00, 45.00]	43.00 [41.00, 45.00]	42.00 [40.00, 44.00]	<0.001
Creatinine, median [IQR]	75.14 [63.65, 87.52]	75.14 [63.65, 87.52]	74.26 [63.65, 88.40]	0.111
Calcium, median [IQR]	2.35 [2.30, 2.40]	2.35 [2.30, 2.40]	2.35 [2.28, 2.40]	0.485
Neutrophil count, median [IQR]	4.00 [3.10, 5.10]	4.00 [3.10, 5.10]	4.00 [3.20, 5.20]	0.002
Monocyte count, median [IQR]	0.50 [0.40, 0.70]	0.50 [0.40, 0.70]	0.60 [0.40, 0.70]	<0.001
Lymphocyte count, median [IQR]	2.00 [1.70, 2.50]	2.10 [1.70, 2.50]	1.90 [1.60, 2.40]	<0.001
White blood cell count, median [IQR]	6.90 [5.70, 8.30]	6.90 [5.70, 8.30]	6.90 [5.70, 8.40]	0.511
Platelet count, median [IQR]	237.00 [203.00, 279.00]	238.00 [204.00, 279.00]	233.00 [196.00, 277.00]	0.004
Inflammatory indicators				
NLR,median [IQR]	1.95 [1.50, 2.55]	1.93 [1.48, 2.51]	2.10 [1.59, 2.80]	<0.001
dNLR, median [IQR]	0.84 [0.80, 0.87]	0.84 [0.80, 0.87]	0.84 [0.80, 0.87]	0.087
MLR, median [IQR]	0.26 [0.21, 0.33]	0.26 [0.21, 0.33]	0.29 [0.23, 0.36]	<0.001
NMLR, median [IQR]	2.22 [1.73, 2.87]	2.20 [1.71, 2.81]	2.40 [1.84, 3.15]	<0.001
SIRI, median [IQR]	1.04 [0.73, 1.50]	1.02 [0.72, 1.47]	1.15 [0.82, 1.68]	<0.001
SII, median [IQR]	462.68 [336.54, 640.64]	458.18 [334.80, 633.32]	492.57 [349.96, 688.03]	<0.001
Monocyte count, median [IQR]	116.67 [93.16, 144.81]	116.11 [92.86, 144.00]	120.53 [95.26, 151.87]	<0.001

^**a**^ Wilcoxon rank-sum teat for complex survey samples; chi-squared test with Rao & Scott’s second-order correction.

BMI, body mass index; SII, Systemic Immune - Inflammation Index; SIRI, Systemic Inflammation Response Index; MLR, Monocyte - to - Lymphocyte Ratio; NMLR, (Neutrophil + Monocyte) - to - Lymphocyte Ratio; NLR, Neutrophil - to - Lymphocyte Ratio; dNLR, Derived Neutrophil - to - Lymphocyte Ratio; PLR, Platelet - to - Lymphocyte Ratio.

### Association between CBC-derived inflammatory indicators and OA

Multivariate logistic regression analysis showed (Table 2) that after full adjustment for covariates in model 3, the five inflammatory indicators SII, SIRI, MLR, NMLR, and NLR were all positively associated with OA, with the following odds ratio (OR) values: SII: OR = 1.000, 95% CI: 1.000–1.000; SIRI: OR = 1.068, 95% CI: 1.107–1.121; MLR: OR = 1.703, 95% CI: 1.161–2.499; NMLR: OR = 1.064, 95% CI: 1.022–1.108; NLR: OR = 1.066, 95% CI: 1.021–1.114. However, no significant association was found between dNLR and OA. Then, we divided the continuous inflammatory indicators into three groups. Participants in the highest tertile of MLR and SIRI had a considerably higher likelihood of having OA than those in the lowest tertile, indicating a strong positive association, according to the fully adjusted model. No positive association was found between the remaining inflammatory indicators in the other tertile models (T2-T3) and the lowest tertile (T1), suggesting that there may be nonlinear associations between these three CBC-derived inflammatory indicators and OA prevalence. We then performed RCS analysis and threshold analysis to test the nonlinear association between these CBC-derived inflammatory indices and OA prevalence.

**TABLE 2:** Weighted multivariable logistic regression analyses for inflammatory indicators and osteoarthritis.

Index	Characteristic	Crude model^a^		Model 1^b^		Model 2^c^		Model 3^d^	
		OR (95% CI)	P value	OR (95% CI)	P value	OR (95% CI)	P value	OR (95% CI)	P value
NLR	Continuous	1.190 (1.144, 1.237)	<0.001***	1.095 (1.053, 1.138)	<0.001***	1.069 (1.024, 1.116)	0.003**	1.066 (1.021, 1.114)	0.004**
	Tertile 1 (1.29 [<1.64])	Reference	​	Reference	​	Reference	​	Reference	​
	Tertile 2 (1.95 [1.64–2.32])	1.202 (1.066, 1.354)	0.003**	1.115 (0.995, 1.250)	0.060	1.052 (0.937, 1.181)	0.389	1.046 (0.932, 1.174)	0.437
	Tertile 3 (2.93 [>2.32])	1.641 (1.446, 1.861)	<0.001***	1.274 (1.101, 1.475)	0.001**	1.146 (0.983, 1.336)	0.081	1.136 (0.975, 1.324)	0.100
	p for trend	​	<0.001***	​	0.002**	​	0.082	​	0.100
dNLR	Continuous	0.622 (0.272, 1.425)	0.259	1.172 (0.463, 2.965)	0.735	0.694 (0.259, 1.864)	0.465	0.643 (0.243, 1.703)	0.369
	Tertile 1 (0.78 [<0.82])	Reference	​	Reference	​	Reference	​	Reference	​
	Tertile 2 (0.84 [0.82–0.86])	0.908 (0.806, 1.022)	0.107	0.911 (0.794, 1.045)	0.180	0.877 (0.761, 1.011)	0.070	0.873 (0.757, 1.006)	0.061
	Tertile 3 (0.88 [>0.86])	0.896 (0.800, 1.004)	0.058	0.962 (0.847, 1.093)	0.549	0.891 (0.781, 1.018)	0.088	0.884 (0.775, 1.008)	0.065
	p for trend	​	0.050	​	0.478	​	0.076	​	0.056
MLR	Continuous	5.777 (3.819, 8.740)	<0.001***	1.797 (1.261, 2.560)	0.001**	1.697 (1.150, 2.504)	0.008**	1.703 (1.161, 2.499)	0.007**
	Tertile 1 (0.18 [<0.23])	Reference	​	Reference	​	Reference	​	Reference	​
	Tertile 2 (0.26 [0.23–0.31])	1.406 (1.231, 1.605)	<0.001***	1.265 (1.086, 1.474)	0.003**	1.284 (1.094, 1.506)	0.003**	1.295 (1.105, 1.517)	0.002**
	Tertile 3 (0.38 [>0.31])	1.897 (1.664, 2.162)	<0.001***	1.340 (1.164, 1.542)	<0.001***	1.350 (1.161, 1.568)	<0.001***	1.353 (1.167, 1.569)	<0.001***
	p for trend	​	<0.001***	​	<0.001***	​	<0.001***	​	<0.001***
NMLR	Continuous	1.186 (1.142, 1.231)	<0.001***	1.090 (1.051, 1.130)	<0.001***	1.066 (1.024, 1.109)	0.002**	1.064 (1.022, 1.108)	0.003**
	Tertile 1 (1.51 [<1.89])	Reference	​	Reference	​	Reference	​	Reference	​
	Tertile 2 (2.22 [1.89–2.61])	1.188 (1.060, 1.331)	0.003**	1.107 (0.994, 1.232)	0.064	1.045 (0.938, 1.164)	0.421	1.039 (0.933, 1.158)	0.479
	Tertile 3 (3.26 [>2.61])	1.677 (1.484, 1.895)	<0.001***	1.278 (1.112, 1.467)	<0.001***	1.154 (0.999, 1.332)	0.052	1.145 (0.993, 1.321)	0.061
	p for trend	​	<0.001***	​	<0.001***	​	0.051	​	0.059
SII	Continuous	1.000 (1.000, 1.001)	<0.001***	1.000 (1.000, 1.000)	<0.001***	1.000 (1.000, 1.000)	0.031**	1.000 (1.000, 1.000)	0.038**
	Tertile 1 (289.25 [<375.66])	Reference	​	Reference	​	Reference	​	Reference	​
	Tertile 2 (469.93 [375.66–573.00])	1.130 (1.011, 1.262)	0.031**	1.006 (0.893, 1.134)	0.916	0.933 (0.820, 1.061)	0.284	0.928 (0.816, 1.056)	0.252
	Tertile 3 (766.11 [>573.00])	1.386 (1.249, 1.538)	<0.001***	1.165 (1.035, 1.312)	0.012	1.013 (0.891, 1.152)	0.844	0.999 (0.880, 1.135)	0.991
	p for trend	​	<0.001***	​	0.007**	​	0.626	​	0.782
SIRI	Continuous	1.242 (1.187, 1.300)	<0.001***	1.139 (1.091, 1.189)	<0.001***	1.068 (1.018, 1.120)	0.007**	1.068 (1.017, 1.121)	0.009**
	Tertile 1 (0.60 [<0.83])	Reference	​	Reference	​	Reference	​	Reference	​
	Tertile 2 (1.04 [0.83–1.31])	1.403 (1.243, 1.584)	<0.001***	1.296 (1.130, 1.486)	<0.001***	1.171 (1.018, 1.347)	0.027**	1.164 (1.011, 1.341)	0.035**
	Tertile 3 (1.80 [>1.31])	1.713 (1.526, 1.922)	<0.001***	1.398 (1.227, 1.592)	<0.001***	1.164 (1.016, 1.334)	0.029**	1.156 (1.010, 1.323)	0.036**
	p for trend	​	<0.001***	​	<0.001***	​	0.076	​	0.089

^a^ No variables were adjusted for the crude model.

^b^ Model 1 was adjusted for age, gender, race, marital status, and education level.

^c^ Model 2 was adjusted for age, gender, race, marital status, education level, smoking status, drinking status, energy intake, BMI, diabetes, and hypertension.

^d^ Model 3 was adjusted for age, gender, race, marital status, education level, smoking status, drinking status, energy intake, BMI, diabetes, hypertension, urine acid, urine creatinine, albumin, and calcium.

### Nonlinear relationship between CBC-derived inflammatory indicators and OA

**FIGURE 2:**
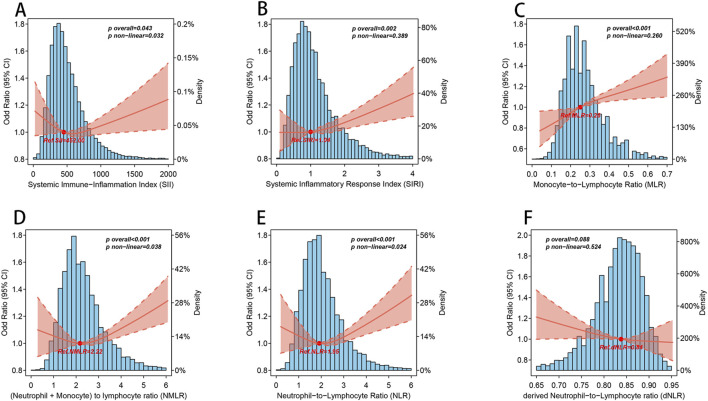
The nonlinear association between CBC-derived inflammatory indicators and OA. Blue histogram bars represent probability density estimates. Solid rad line represents the smooth curve fit between CBC-derived inflammatory indicators and OA. Pale red bands between dashed lines represent the 95% of confidence interval from the fit. The above models were adjusted for age, gender, race, marital status, education level, smoking status, drinking status, energy intake, BMI, diabetes, hypertension, urine acid, urine creatinine, albumin, and calcium. **(A)** SII and OA; **(B)** SIRI and OA; **(C)** MLR and OA; **(D)** NMLR and OA; **(E)** NLR and OA; **(F)** dNLR and OA.

As shown in Figure 2, we ultilized three nodes (10th, 50th, and 90th) and drew the RCS curve with the median as the reference value The RCS image showed that, except for dNLR, there was a significant overall trend between the other CBC-derived inflammatory indicators and the prevalence of OA (p for overall <0.05), among which SII, NLR, and NMLR showed significant nonlinear associations (p for nonlinear <0.05). Consistent with the results of the weighted logistic regression analysis, MLR and SIRI showed significant linear associations (p for nonlinear >0.05).

It is worth noting that when we performed threshold analysis of the five CBC-derived inflammatory indicators associated with OA prevalence (Table 3), the nonlinear association pattern of SII differed from those of NLR and NMLR. Specifically, the dose-response pattern between SII and the prevalence of OA was “U”-shaped, while the dose-response pattern between NLR and NMLR and the prevalence of OA was “J”-shaped, and the log-likelihood ratio test p < 0.05. For SII, the estimated threshold was 402.857. When SII exceeded this level, OA prevalence increased significantly, whereas values below the threshold showed a decreasing trend. Similarly, a threshold of 2.522 was identified for NLR. Below this threshold, the association between NLR and OA was not statistically significant, whereas above it the risk of OA increased markedly, with an adjusted OR of 1.093 (1.047–1.141) per unit increase. NMLR also showed a comparable J-shaped pattern, with a threshold of 1.538. Beyond this threshold, the risk of OA rose significantly, with an adjusted OR of 1.070 (1.036–1.104).

**TABLE 3:** Analysis of the threshold effect between inflammatory indicators and osteoarthritis.

Outcome:OA	NLR	MLR	NMLR	SII	SIRI
	OR (95%CI)	OR (95%CI)	OR (95%CI)	OR (95%CI)	OR (95%CI)
Fitting by standard linear model					
OR	1.058 (1.024–1.093)	1.930 (1.407–2.639)	1.057 (1.026–1.09)	1.000 (1.000–1.000)	1.079 (1.032–1.127)
P-value	0.001	<0.001	<0.001	0.053	0.001
Fitting by two-piecewise linear model					
Breakpoint(K)	2.522	0.143	1.538	402.857	0.632
OR1 < K	0.968 (0.891–1.052)	0.017 (0.000–4.789)	0.777 (0.586–1.038)	0.999 (0.999–1.000)	0.656 (0.385–1.131)
	0.447	0.144	0.084	0.019	0.126
OR2 > K	1.093 (1.047–1.141)	2.042 (1.48–2.811)	1.07 (1.036–1.104)	1.000 (1.000–1.000)	1.092 (1.043–1.143)
	<0.001	<0.001	<0.001	0.012	<0.001
Logarithmic likelihood ratio test P-value	0.023	0.098	0.036	0.008	0.072

Age, gender, race, marital status, education level, smoking status, drinking status, energy intake, BMI, diabetes, hypertension, urine acid, urine creatinine, albumin,and calcium were adjusted.

In the threshold analysis, the log-likelihood ratio test of the two-segment linear regression model of MLR and SIRI was p > 0.05, which once again verified the strong positive association between MLR and SIRI and the prevalence of OA.

### Subgroup and sensitivity analyses between CBC-derived inflammatory indicators and OA

**FIGURE 3:**
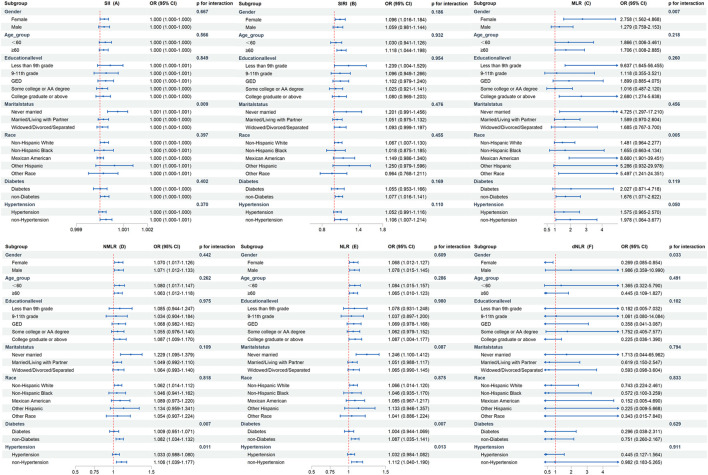
Subgroup analysis of the association between CBC-derived inflammatory indicators and OA prevalence. The above models were adjusted for age, gender, race, marital status, education level, smoking status, drinking status, energy intake, BMI, diabetes, hypertension, urine acid, urine creatinine, albumin, and calcium. In each case, the models were not adjusted for stratification variables. **(A)** SII and OA; **(B)** SIRI and OA; **(C)** MLR and OA; **(D)** NMLR and OA; **(E)** NLR and OA; **(F)** dNLR and OA.

In certain categories, there was inconsistent evidence of a correlation between elevated CBC-derived inflammatory indicators and OA incidence (Figure 3). The findings demonstrated that most subgroups did not exhibit a substantially distinct association between CBC-derived inflammatory indicators and OA incidence, and all interaction p-values were greater than 0.05. However, we found that increased MLR and NMLR were associated with increased OA prevalence in non-diabetic and non-hypertensive populations after adjusting for all covariates, but not in diabetic and hypertensive populations.

To test the robustness of the study results, we performed sensitivity analyses (Table 4). Multiple imputation was performed for variables with missing values. Then, the association between CBC-derived inflammatory indicators and OA prevalence was repeatedly verified in 10 imputed complete data sets. The impact of missing data on the results was somewhat mitigated by the consistent association between OA prevalence and CBC-derived inflammatory indicators throughout the 10 imputed data sets.

**TABLE 4:** Weighted multivariable logistic regression analyses for inflammatory indicators and osteoarthritis after multiple imputation.

Index	Characteristic	Crude model^a^		Model 1^b^		Model 2^c^		Model 3^d^	
		OR (95% CI)	P value	OR (95% CI)	P value	OR (95% CI)	P value	OR (95% CI)	P value
NLR	Continuous	1.190 (1.156, 1.225)	<0.001***	1.082 (1.050, 1.116)	<0.001***	1.058 (1.024, 1.093)	<0.001***	1.060 (1.026, 1.095)	<0.001***
	Tertile 1 (1.32 [<1.65])	Reference	​	Reference	​	Reference	​	Reference	​
	Tertile 2 (1.95 [1.65–2.32])	1.188 (1.092, 1.292)	<0.001***	1.078 (0.992, 1.171)	0.076	1.014 (0.930, 1.105)	0.755	1.014 (0.930, 1.105)	0.752
	Tertile 3 (2.91 [>2.32])	1.660 (1.511, 1.823)	<0.001***	1.268 (1.141, 1.410)	0.001**	1.129 (1.011, 1.260)	0.031*	1.131 (1.013, 1.264)	0.029*
	p for trend	​	<0.001***	​	<0.001***	​	0.023*	​	0.021*
dNLR	Continuous	0.818 (0.465, 1.438)	0.483	1.559 (0.806, 3.013)	0.185	0.944 (0.469, 1.900)	0.870	0.907 (0.452, 1.818)	0.781
	Tertile 1 (0.78 [<0.82])	Reference	​	Reference	​	Reference	​	Reference	​
	Tertile 2 (0.84 [0.82–0.86])	0.990 (0.910, 1.078)	0.820	1.011 (0.915, 1.117)	0.825	0.979 (0.884, 1.084)	0.676	0.976 (0.881, 1.082)	0.644
	Tertile 3 (0.88 [>0.86])	0.932 (0.857, 1.013)	0.097	1.018 (0.924, 1.121)	0.721	0.946 (0.857, 1.044)	0.264	0.941 (0.852, 1.038)	0.223
	p for trend	​	0.113	​	0.721	​	0.278	​	0.236
MLR	Continuous	5.920 (4.452, 7.872)	<0.001***	1.655 (1.248, 2.194)	<0.001***	1.611 (1.192, 2.177)	0.002**	1.677 (1.241, 2.267)	<0.001***
	Tertile 1 (0.18 [<0.22])	Reference	​	Reference	​	Reference	​	Reference	​
	Tertile 2 (0.26 [0.22–0.30])	1.296 (1.163, 1.444)	<0.001***	1.133 (1.011, 1.271)	0.032*	1.147 (1.021, 1.290)	0.022*	1.153 (1.027, 1.295)	0.017*
	Tertile 3 (0.38 [>0.30])	1.832 (1.679, 1.999)	<0.001***	1.235 (1.123, 1.357)	<0.001***	1.247 (1.130, 1.378)	<0.001***	1.257 (1.139, 1.388)	<0.001***
	p for trend	​	<0.001***	​	<0.001***	​	<0.001***	​	<0.001***
NMLR	Continuous	1.187 (1.155, 1.221)	<0.001***	1.078 (1.048, 1.109)	<0.001***	1.056 (1.024, 1.088)	<0.001***	1.058 (1.027, 1.091)	<0.001***
	Tertile 1 (1.53 [<1.90])	Reference	​	Reference	​	Reference	​	Reference	​
	Tertile 2 (2.22 [1.90–2.60])	1.187 (1.091, 1.291)	<0.001***	1.077 (0.990, 1.171)	0.083	1.024 (0.937, 1.119)	0.599	1.025 (0.938, 1.119)	0.585
	Tertile 3 (3.25 [>2.60])	1.695 (1.548, 1.857)	<0.001***	1.257 (1.136, 1.390)	<0.001***	1.130 (1.018, 1.255)	0.022*	1.134 (1.021, 1.259)	0.019*
	p for trend	​	<0.001***	​	<0.001***	​	0.018*	​	0.015*
SII	Continuous	1.000 (1.000, 1.000)	<0.001***	1.000 (1.000, 1.000)	0.001**	1.000 (1.000, 1.000)	0.188	1.000 (1.000, 1.000)	0.157
	Tertile 1 (289.25 [<380.16])	Reference	​	Reference	​	Reference	​	Reference	​
	Tertile 2 (469.93 [380.16–585.57])	1.163 (1.064, 1.270)	<0.001***	1.063 (0.974, 1.160)	0.171	1.000 (0.914, 1.094)	0.995	0.999 (0.914, 1.093)	0.986
	Tertile 3 (766.11 [>585.57])	1.350 (1.238, 1.472)	<0.001***	1.149 (1.046, 1.262)	0.004**	1.004 (0.909, 1.109)	0.937	1.005 (0.911, 1.108)	0.927
	p for trend	​	<0.001***	​	0.005**	​	0.931	​	0.916
SIRI	Continuous	1.237 (1.196, 1.280)	<0.001***	1.117 (1.077, 1.159)	<0.001***	1.060 (1.019, 1.102)	0.004**	1.064 (1.022, 1.107)	0.003**
	Tertile 1 (0.65 [<0.83])	Reference	​	Reference	​	Reference	​	Reference	​
	Tertile 2 (1.03 [0.83–1.31])	1.188 (1.092, 1.292)	<0.001***	1.078 (0.992, 1.171)	0.076	1.014 (0.930, 1.105)	0.755	1.014 (0.930, 1.105)	0.752
	Tertile 3 (1.68 [>1.31])	1.660 (1.511, 1.823)	<0.001***	1.268 (1.141, 1.410)	<0.001***	1.129 (1.011, 1.260)	0.031*	1.131 (1.013, 1.264)	0.029*
	p for trend	​	<0.001***	​	<0.001***	​	0.022*	​	0.020*

^a^ No variables were adjusted for the crude model.

^b^ Model 1 was adjusted for age, gender, race, marital status, and education level.

^c^ Model 2 was adjusted for age, gender, race, marital status, education level, smoking status, drinking status, energy intake, BMI, diabetes, and hypertension.

^d^ Model 3 was adjusted for age, gender, race, marital status, education level, smoking status, drinking status, energy intake, BMI, diabetes, hypertension, urine acid, urine creatinine, albumin, and calcium.

### Establishment of OA prediction model

**FIGURE 4:**
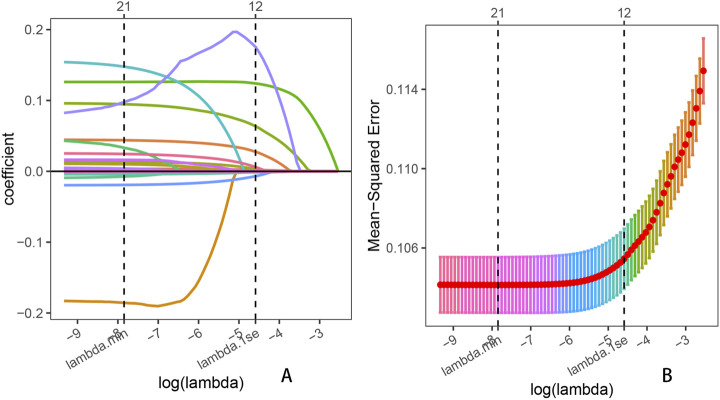
Lasso penalized regression analysis was used to identify key predictors of OA prevalence. **(A)** Coefficient shrinkage for all 25 variables, including age, sex, race, education level, marital status, diabetes, hypertension, BMI, smoking status, drinking status, daily energy intake, uric acid, albumin, total calcium, neutrophil count, lymphocyte count, leukocyte count, monocyte count, platelet count, NLR, dNLR, MLR, NMLR, SIRI, and SII; **(B)** Ten-fold cross-validation for the LASSO regression.

A total of 25 potential predictor variables, identified from the literature and clinical experience, were incorporated into the LASSO regression analysis. After 10-fold cross-validation, we obtained the minimum lambda value (λ_min_ = 0.000392) and the lambda value under 1 standard error (λ_1se_ = 0.010160). To simplify the prediction model as much as possible, we selected λ_1se_ as the optimal penalty parameter. According to the minimum criterion of non-zero coefficients, 12 variables were ultimately retained (Figure 4). These 12 variables included sex, education level, marital status, diabetes, hypertension, smoking status, BMI, daily energy intake, albumin, platelet count, MLR, and NMLR. Results from the univariable and multivariable logistic regression analyses indicated that all osteoarthritis-related risk factors were statistically significant (p < 0.05). Detailed regression results are provided in Supplementary Table S4.

Subsequently, considering the feasibility of data collection in primary healthcare settings, we excluded albumin, daily energy intake, and smoking status, as these factors are not routinely accessible through standard physical examinations, brief patient interviews, or complete blood count testing. To protect patient privacy, we also removed education level and marital status.

**FIGURE 5:**
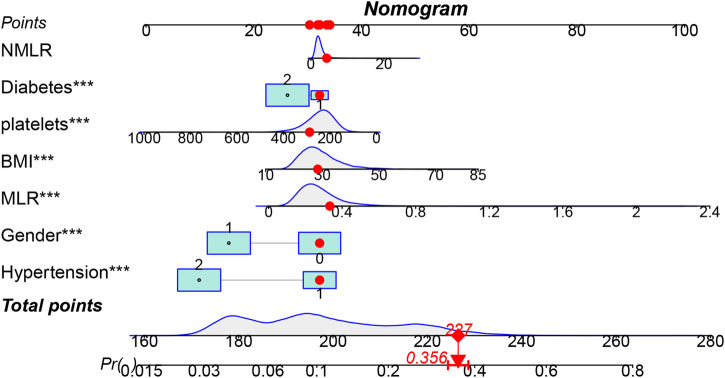
Visualization of OA prevalence risk model. The top horizontal line of the column bar is the score bar, and the sum of the scores is the risk of osteoarthritis. A total of 7 indicators were included in the risk prediction table.

To provide doctors with a simple, understandable, and easy-to-use visual scoring tool, seven predictors—sex, diabetes, hypertension, BMI, platelet count, MLR, and NMLR—were selected and incorporated into the final nomogram (Figure 5). In this nomogram, each predictor is assigned a corresponding number of points, reflecting its relative contribution to OA risk. The total score obtained by summing these points provides an overall risk estimate, from which the probability of OA can be directly calculated.

**FIGURE 6:**
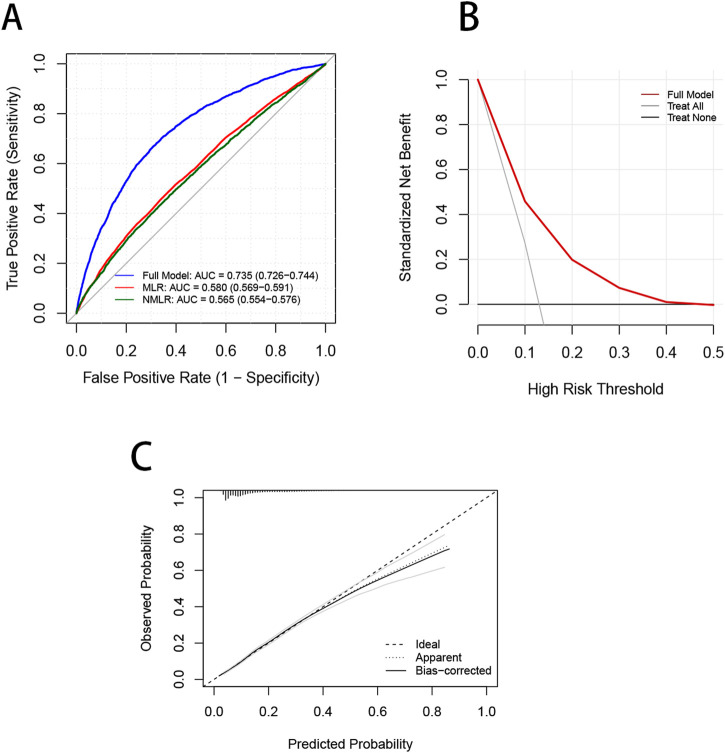
Validation of the OA prevalence risk model. **(A)** ROC curves are used to evaluate the ability of the Nomagram model and CBC inflammation-derived indicators to predict OA prevalence. **(B)** The red curve in the DCA curve represents the net benefit of the model, and the black lines represent the benefits of “no intervention” and “full intervention”, respectively. **(C)** The dotted line in the calibration curve represents that the model’s predicted probability is completely consistent with the actual observed probability, the dotted line reflects the model’s prediction performance without bias correction, and the actual line represents the model’s prediction performance after bias correction using bootstrap resampling.

To evaluate the predictive performance of the CBC-derived inflammatory indicators model and the OA prediction model, the respective receiver operating characteristic (ROC), decision curve (DCA), and calibration curves were plotted (Figure 6). The AUC of the whole prediction model was 0.735 (95% CI: 0.726–0.744), with a sensitivity of 73.4% and a specificity of 67.5%. The AUCs of MLR and NMLR were 0.580 (95% CI: 0.569–0.591) and 0.565 (95% CI: 0.554–0.576), respectively. To further evaluate the model’s robustness, we performed internal validation using bootstrap resampling. After 1,000 resamplings, the model showed an accuracy of 0.867 and a kappa of 0.317, indicating good accuracy and moderate agreement. We also generated ROC curves based on the bootstrap samples (Supplementary Figure S3), which illustrate both the model’s predictive performance and the stability of the internal validation results. Additionally, the DCA curve demonstrated that the model’s net benefit outperformed the “all-cure” and “no-cure” approaches across a broad range of threshold probabilities and had substantial clinical relevance. Although the calibration curve was somewhat below the optimal value at high risks, it was frequently around the reference line, particularly with the best match at low and medium risks.

## Discussion

Utilizing data from the National Health and Nutrition Examination Survey (NHANES) from 2007 to 2020, the current study investigated the association between the prevalence of OA in Americans and CBC-derived inflammatory indicators. 3,195 (13.25%) of the 24,112 individuals in the final sample had an OA diagnosis. Weighted multivariate logistic regression models found that increased OA incidence was associated with increased levels of SII, SIRI, MLR, NMLR, and NLR. The crude model and the modified models 1, 2, and 3 all showed this connection to be consistent. Stratifying CBC-derived inflammatory indicators into tertiles, we found a strong linear association between SIRI and MLR and the prevalence of OA. This strong linear association was further confirmed by RCS and threshold analyses, which also revealed potential nonlinear associations between SII, NMLR, and NLR and OA prevalence. Subgroup analyses and interaction tests showed stratification by diabetes status and hypertension.

SIRI and MLR are comprehensive indicators closely related to systemic inflammation and the immune response. These ratios have been widely studied in various inflammatory-related diseases, including psoriasis, cardiovascular disease, and cancer [23–25]. In a previous study, He et al. found that the risk of OA increased by 15% for each unit increase in log 2 (SIRI). However, in a sensitivity analysis that changed log 2 (SIRI) from a continuous variable to a categorical variable (quartiles), no positive correlation was found between the other quartile models (Q2-Q4) and the lowest quartile (Q1) [17]. In contrast, Yan et al. demonstrated that SIRI was significantly positively correlated with OA. In all adjusted models, the prevalence of OA gradually increased with increasing SIRI, especially in Q3 and Q4 [26]. In the present study, we found a strong linear association between MLR (OR = 1.35 [1.17, 1.57] T3 vs. T1; OR = 1.30 [1.11, 1.52] T2 vs. T1) and SIRI (OR = 1.16 [1.01, 1.52] T3 vs. T1; OR = 1.16 [1.01, 1.34] T2 vs. T1) and the prevalence of OA, supporting their findings.

Our study also explored the association between OA and other inflammatory indices, SII, NLR, and NMLR. Although the logistic regression model with SII, NLR, and NMLR as continuous variables showed a significant association between higher levels of these indices and increased OA prevalence, we found no significant association between the other tertiles (T2–T3) and the lowest tertile (T1) after transforming them into tertiles. Therefore, we inferred that there might be nonlinear associations between SII, NLR, and NMLR and OA prevalence. The subsequent RCS models confirmed this nonlinear pattern, and the threshold analysis further identified their risk thresholds.

These risk thresholds are not intended to serve as diagnostic cut-off values. Instead, they offer practical reference points for understanding how inflammatory indicators, even within the range observed in healthy adults, may indicate varying risk trends. The 95% reference interval for SII reported by Liu et al. is 162–811 [27], and the risk threshold identified in this study (SII = 402.857) falls within this range for healthy populations. Clinically, this may prompt physicians to exercise greater vigilance in identifying patients in subclinical or early stages of OA. When an individual’s SII level deviates significantly from the lowest-risk point, clinicians may consider more cautious evaluations, such as follow-up or imaging assessments, even if the value remains within the normal range. NLR has been extensively studied in cardiovascular diseases, infections, inflammatory disorders, and various cancers. The NLR threshold (2.522) identified in our study for OA risk also lies within the established normal range of 0.78–3.53 [28]. When a patient’s NLR exceeds this threshold, clinicians may be prompted to pay closer attention to subtle joint symptoms, medical history, obesity, and other OA-related risk factors, enabling earlier intervention when appropriate. As for NMLR, a relatively novel inflammatory indicator, its reference interval has yet to be determined. Nevertheless, we hypothesize that its risk pattern may resemble that of NLR. In summary, these risk thresholds can assist clinicians in understanding how different levels of inflammatory indicators correspond to changes in OA risk among otherwise healthy individuals, thereby supporting earlier identification and timely clinical management.

For the first time, we studied the association between NMLR and dNLR and OA prevalence. NMLR and dNLR are considered novel indicators of cellular immune activation and have attracted the attention of many researchers [29, 30]. These two indices reflect interactions among neutrophils, monocytes/macrophages, and lymphocytes. They may offer fresh perspectives on the intricate association between inflammatory processes and osteoarthritis. In this study, both the continuous and tertile models showed that the association between dNLR and OA was not statistically significant.

Subgroup analyses revealed a consistent pattern that higher MLR/NMLR levels were significantly associated with increased OA risk among individuals without diabetes or hypertension. In contrast, this association did not reach statistical significance among those with either condition. One possible explanation is that, in individuals with diabetes or hypertension, systemic inflammation may contribute to OA development primarily through these comorbidities rather than acting independently. Insulin resistance—a common feature of both diabetes and hypertension—has recently been recognized as a key risk factor underlying these disorders [31]. Under systemic inflammatory conditions, elevated cytokine production by circulating immune cells can impair insulin signaling through multiple intermediate pathways [32]. For example, tumor necrosis factor-α (TNF-α) activates the IKKβ, SOCS1/3, PKC, and ERK pathways, leading to serine phosphorylation of insulin receptor substrates (IRS) in insulin-responsive tissues. These pathways suppress Akt activity, a critical mediator of the insulin signaling cascade [33–35]. The resulting impairment of insulin signaling may further amplify systemic inflammation and contribute to OA pathogenesis among individuals with diabetes or hypertension. Consequently, the independent associations between these inflammatory indices and OA risk may be attenuated in these subgroups.

Our findings show that MLR, SIRI, and the threshold-based increases in NLR, MLR, and NMLR are positively associated with OA risk. These composite inflammatory indices integrate circulating counts of platelets, neutrophils, monocytes, and lymphocytes, collectively reflecting the overall level of systemic inflammation. Systemic inflammatory states associated with obesity, aging, or chronic diseases may promote local joint inflammation [36, 37]. Elevated systemic inflammation leads to increased recruitment of immune cells, including neutrophils and monocytes/macrophages, into the joint cavity [38, 39]. These infiltrating cells release large amounts of pro-inflammatory cytokines, such as IL-1β, IL-6, and TNF-α [8], thereby directly contributing to and amplifying intra-articular inflammation.

By activating the mitogen-activated protein kinase (MAPK) signaling pathway, IL-1β induces catabolic processes, including cartilage degradation, which are key mechanisms in OA progression [40]. IL-1β activates MAPK signaling, which upregulates catabolic enzymes, including MMP-1, MMP-3, MMP-13, ADAMTS-4, and ADAMTS-5. These enzymes then directly degrade components of the extracellular matrix. These processes lead to chondrocyte hypertrophy, dedifferentiation, and ultimately apoptosis [41]. Another major pathway in IL-1β–mediated OA progression is NF-κB. Once activated, NF-κB suppresses type II collagen synthesis and increases the production of matrix metalloproteinases, aggrecanases, and various chemokines—including IL-8, monocyte chemoattractant protein-1 (MCP-1/CCL2), CCL5, and macrophage inflammatory protein-1α (MIP-1α)—which further attract inflammatory cells and intensify joint inflammation [42].

An important finding of our study is that, compared with indices incorporating only monocytes/macrophages (MLR), those incorporating neutrophils (NLR, NMLR) showed clear nonlinear relationships and threshold effects. This pattern suggests that different immune cell populations may have distinct associations with OA risk. One possible explanation is that neutrophils may exert certain protective effects against cartilage damage under conditions of low-grade systemic inflammation [43]. Previous studies have shown that neutrophil-derived extracellular vesicles (EVs) can act in an anti-inflammatory manner [44] and protect cartilage by increasing type II collagen and reducing type X collagen within the joint. Further evidence indicates that neutrophil EVs enhance cartilage protection by inducing transforming growth factor-β (TGF-β) production, a key mediator of chondrocyte homeostasis. This process promotes the deposition of type II collagen (COL2) and glycosaminoglycans (GAGs), while downregulating cartilage-degrading enzymes (MMPs) [45].

We also observed a similar nonlinear pattern when comparing indices involving neutrophils and platelets (SII) with those involving neutrophils and monocytes/macrophages (SIRI). Platelet-rich plasma (PRP) has been shown to have beneficial effects in OA [46], yet the specific role of platelets and their underlying mechanisms remains incompletely understood. In rodent models of OA, platelet-derived ADP has been shown to increase bone morphogenetic protein 7 (BMP7) levels markedly. Through autocrine and paracrine actions, BMP7 promotes chondrocyte proliferation via the ERK/CDK1/cyclin B1 signaling pathway [47]. The protective effects of platelets on cartilage may help explain the inverse association observed between SII and OA risk below the identified threshold.

This study comprehensively explored the association between CBC-derived inflammatory indicators and OA prevalence. After observational analysis, we developed and validated a prediction model incorporating the CBC-derived inflammatory indicators and demonstrated its clinical utility. After LASSO regression analysis, we identified seven independent risk factors with the best predictive power for OA, including MLR and NMLR, and demonstrated their clinical utility. In addition, it should be clarified that the proposed model is particularly applicable to OA screening in primary healthcare settings, where access to advanced imaging modalities may be limited and CBC-derived inflammatory indicators are readily available.

The present research has several significant advantages. Firstly, to fully examine the association between exposure and outcomes, we have used large samples and long-term tracking data from NHANES. Secondly, there is little evidence from earlier research linking CBC-derived inflammatory indicators to OA. Novel quantitative associations between CBC-derived inflammatory indicators and the prevalence of OA among the United States general population are revealed in the present study. Finally, utilizing complete blood counts, one of the most widely used assays in clinical practice, we assessed the association between various inflammatory indicators and the prevalence of OA. These indicators, however, have not been thoroughly examined in earlier research and are often examined as individual indicators. In the present study, we analyzed the indicators in greater detail and comprehensively examined their association with OA prevalence.

However, we must acknowledge some limitations of the present study. Firstly, we could not establish a causal association between CBC-derived inflammatory indicators and OA prevalence due to the cross-sectional design of NHANES. Future population-based prospective cohort studies should be conducted to verify the causal relationship between CBC-derived inflammatory indicators and OA and to clarify their influence on OA development and progression. Secondly, residual confounding persists even after we carefully account for several factors associated with the outcome. It is impossible to completely rule out the possibility that unmeasured variables might influence our findings, and residual confounding could introduce some bias. Thirdly, the NHANES statistics, which are representative of the United States population, served as the basis for the research population. It’s possible that the results won’t apply to groups with distinct healthcare or demographic systems. Additionally, the NHANES database lacks information on OA anatomical sites, longitudinal CBC data, and joint fluid biomarkers that reflect local inflammatory activity. Future studies could focus on addressing these data gaps to explore whether CBC-derived inflammatory indicators exhibit different associations across specific OA sites. Additionally, prospective cohort studies could be conducted, or investigations into the relationship between systemic inflammation and local intra-articular inflammation could be pursued, providing deeper mechanistic insights into how systemic inflammation influences OA progression.

The reliance on self-reported OA diagnoses may introduce several types of information bias. Previous studies have shown that differences in survey methods, question wording, and sample composition can lead to variations of 2–3 percentage points in the prevalence of arthritis based on self-reports [48]. The agreement between large surveys can be as low as 70% [49, 50]. In this context, the self-reported OA prevalence in NHANES is also lower than that in other databases (such as CCS and NADW) that rely on ICD-9-CM diagnostic codes [51]. This suggests that self-reporting may lead to under-ascertainment and affect prevalence estimates. In addition, self-reports are influenced by the intermittent nature of OA symptoms. Individuals are more likely to recall and report a diagnosis when symptoms are pronounced or when they have recently sought medical care. This recall bias may either inflate or attenuate the observed associations between inflammatory indices and OA. Overall, recall bias in self-reported OA may weaken true associations. Therefore, our findings should be interpreted with caution, and future studies using imaging findings or clinical diagnoses are needed to improve the accuracy of outcome definitions.

## Conclusion

In conclusion, in this nationally representative US sample, CBC-derived inflammatory indicators (SII, SIRI, MLR, NMLR, NLR) were significantly associated with OA prevalence. Further investigation revealed that NLR, NMLR, and SII were nonlinearly associated with OA prevalence. Our study reinforces the view that inflammation promotes the pathological process of OA. In addition, we constructed a prediction model for OA risk, emphasizing the predictive power of MLR and NMLR.

## Data Availability

The original contributions presented in the study are included in the article/Supplementary Material, further inquiries can be directed to the corresponding authors. Online repositories include the datasets used in this investigation. Publicly accessible datasets used in the present research are available here: https://www.cdc.gov/nchs/nhanes/.

## Footnotes

1
https://www.cdc.gov/nchs/nhanes
