# Supplementary material for: Inflammatory indicators derived from complete blood counts in relation to osteoarthritis prevalence: findings from the NHANES 2007–2020 cross-sectional survey
Source: Exp Biol Med (Maywood). 2026 Jan 13;250:10815. doi: 10.3389/ebm.2025.10815 (PMC12834830; doi:10.3389/ebm.2025.10815)
Supplement: Supplementary file 2 [file Table1.docx]

**Supplementary Table 1. Variance Inflation Factors for All Variables in the Fully Adjusted Logistic Regression Model**

|  | **SII** | **SIRI** | **MLR** | **NMLR** | **NLR** | **dNLR** |
| --- | --- | --- | --- | --- | --- | --- |
| **Indicator itself** | 1.017 | 1.049 | 1.108 | 1.036 | 1.029 | 1.040 |
| **Age** | 1.117 | 1.721 | 1.465 | 1.544 | 1.395 | 1.456 |
| **Gender** | 1.044 | 1.928 | 1.619 | 1.706 | 1.546 | 1.647 |
| **Race** | 1.119 | 1.276 | 1.075 | 1.199 | 1.088 | 1.131 |
| **Education level** | 1.067 | 1.261 | 1.051 | 1.126 | 1.030 | 1.082 |
| **Marital status** | 1.058 | 1.402 | 1.157 | 1.246 | 1.137 | 1.919 |
| **Hypertension** | 1.088 | 1.604 | 1.340 | 1.434 | 1.312 | 1.375 |
| **Diabetes** | 1.071 | 1.461 | 1.168 | 1.282 | 1.161 | 1.225 |
| **Smoking status** | 1.051 | 1.303 | 1.097 | 1.170 | 1.079 | 1.124 |
| **Drinking status** | 1.036 | 1.258 | 1.046 | 1.115 | 1.026 | 1.067 |
| **Energy intake** | 1.077 | 1.422 | 1.189 | 1.276 | 1.169 | 1.223 |
| **BMI** | 1.121 | 1.693 | 1.371 | 1.502 | 1.323 | 1.412 |
| **Uric_acid** | 1.090 | 1.804 | 1.470 | 1.594 | 1.427 | 1.514 |
| **Creatinine** | 1.256 | 1.937 | 1.332 | 1.479 | 1.313 | 1.379 |
| **Calcium** | 1.201 | 1.681 | 1.399 | 1.513 | 1.369 | 1.444 |
| **Aibumin** | 1.155 | 2.055 | 1.686 | 1.823 | 1.643 | 1.733 |

**Supplementary Table 2. Akaike Information Criterion Values for Fully Adjusted Restricted Cubic Spline Models.**

|  | **3 knots** | **4 knots** | **5 konts** |
| --- | --- | --- | --- |
| SII | **14982.53** | 14982.69 | 14983.01 |
| SIRI | **14978.78** | 14980.35 | 14978.96 |
| MLR | 14972.61 | 14973.36 | **14972.14** |
| NMLR | **14973.75** | 14975.47 | 14977.02 |
| NLR | **14974.29** | 14976.22 | 14977.95 |
| dNLR | 14985.94 | **14982.90** | 14984.16 |

**Supplementary Table 3. Variance Inflation Factors of All Predictors in the Final Predictive**

|  | **VIF** |
| --- | --- |
| **Gender** | 1.223 |
| **Hypertension** | 1.257 |
| **Diabetes** | 1.253 |
| **BMI** | 1.267 |
| **Platelets** | 1.316 |
| **MLR** | 2.445 |
| **NMLR** | 1.990 |

**Supplementary Table 4. Associations Between Candidate Prediction Factors and Osteoarthritis in Logistic Regression Analyses**

| **characteristic** | **Univariable** | |  | **Multivariable** | |
| --- | --- | --- | --- | --- | --- |
|  | **OR(95% CI)** | **P value** |  | **OR(95% CI)** | **P value** |
| **Gender** |  |  |  |  |  |
| Male | Reference |  |  | Reference |  |
| Female | 2.100(1.911,2.037) | **<0.001***** |  | 2.422(2.155,2.723) | **<0.001***** |
| **Education level** |  |  |  |  |  |
| Less than 9th grade | Reference |  |  | Reference |  |
| 9-11th grade | 1.175(0.913,1.512) | 0.209 |  | 1.203(0.920,1.572) | 0.174 |
| High school/GED | 1.386(1.064,1.803) | **0.016*** |  | 1.522(1.176,1.971) | **0.002**** |
| College or AA | 1.439(1.135,1.825) | **0.003**** |  | 1.562(1.220,2.001) | **<0.001***** |
| College above | 1.313(1.014,1.701) | **0.039** |  | 1.807(1.384,2.359) | **<0.001***** |
| **Marital status** |  |  |  |  |  |
| Married | Reference |  |  | Reference |  |
| Widowed | 1.870(1.651,2.117) | **<0.001***** |  | 1.361(1.187,1.561) | **<0.001***** |
| Never married | 0.286(0.234,0.350) | **<0.001***** |  | 0.378(0.307,0.467) | **<0.001***** |
| **Smoking status** |  |  |  |  |  |
| No | Reference |  |  | Reference |  |
| Mild | 2.069(1.811,2.362) | **<0.001***** |  | 1.895(1.635,2.195) | **<0.001***** |
| Intense | 1.023(0.870,1.204) | 0.777 |  | 1.292(1.090,1.531) | **0.004**** |
| **Hypertension** |  |  |  |  |  |
| Yes | Reference |  |  | Reference |  |
| No | 0.268(0.239,0.301) | **<0.001***** |  | 0.363(0.319,0.413) | **<0.001***** |
| **Diabetes** |  |  |  |  |  |
| Yes | Reference |  |  | Reference |  |
| No | 0.402(0.354,0.457) | **<0.001***** |  | 0.729(0.631,0.843) | **<0.001***** |
| **Energy intake** | 1.000(1.000,1.000) |  |  | 1.000(1.000,1.000) | **<0.001***** |
| **BMI** | 1.043(1.036,1.051) | **<0.001***** |  | 1.027(1.018,1.035) | **<0.001***** |
| **Albumin** | 0.910(0.896,0.924) | **<0.001***** |  | 0.975(0.958,0.992) | **<0.001***** |
| **Platelets** | 0.999(0.998,1.000) | **<0.001***** |  | 0.998(0.997,0.999) | **<0.001***** |
| **MLR** | 5.777(3.819,8.740) | **<0.001***** |  | 3.375(1.766,6.452) | **<0.001***** |
| **NMLR** | 1.186(1.142,1.231) | **<0.001***** |  | 1.053(1.023,1.126) | **0.037*** |

*
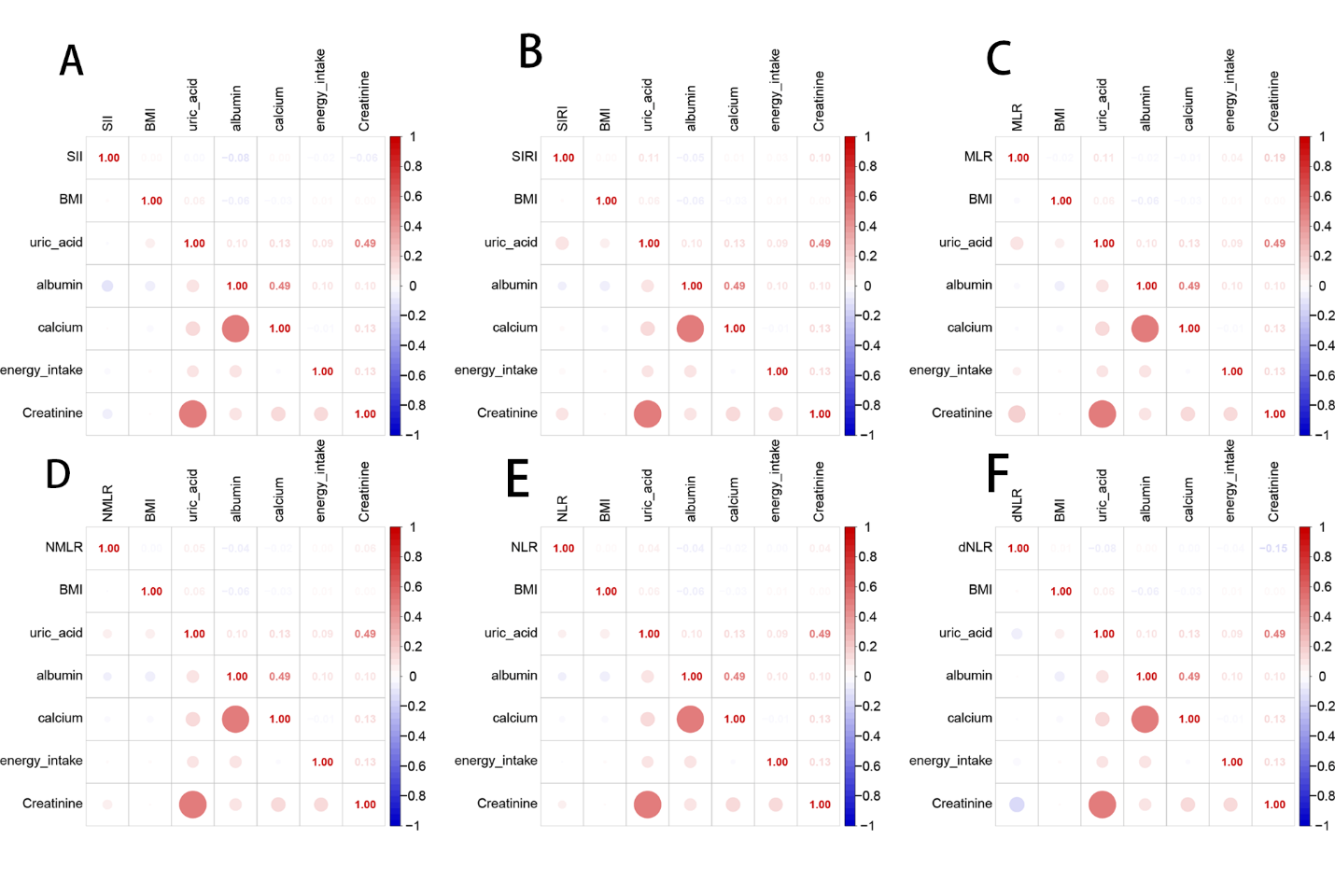
*

**Supplementary Figure 1. Correlation Matrix of Continuous Variables in the Fully Adjusted Model. (A) SII fully adjusted model; (B) SIRI fully adjusted model; (C) MLR fully adjusted model; (D) NMLR fully adjusted model; (E) NLR fully adjusted model; (F) dNLR fully adjusted model.**

*
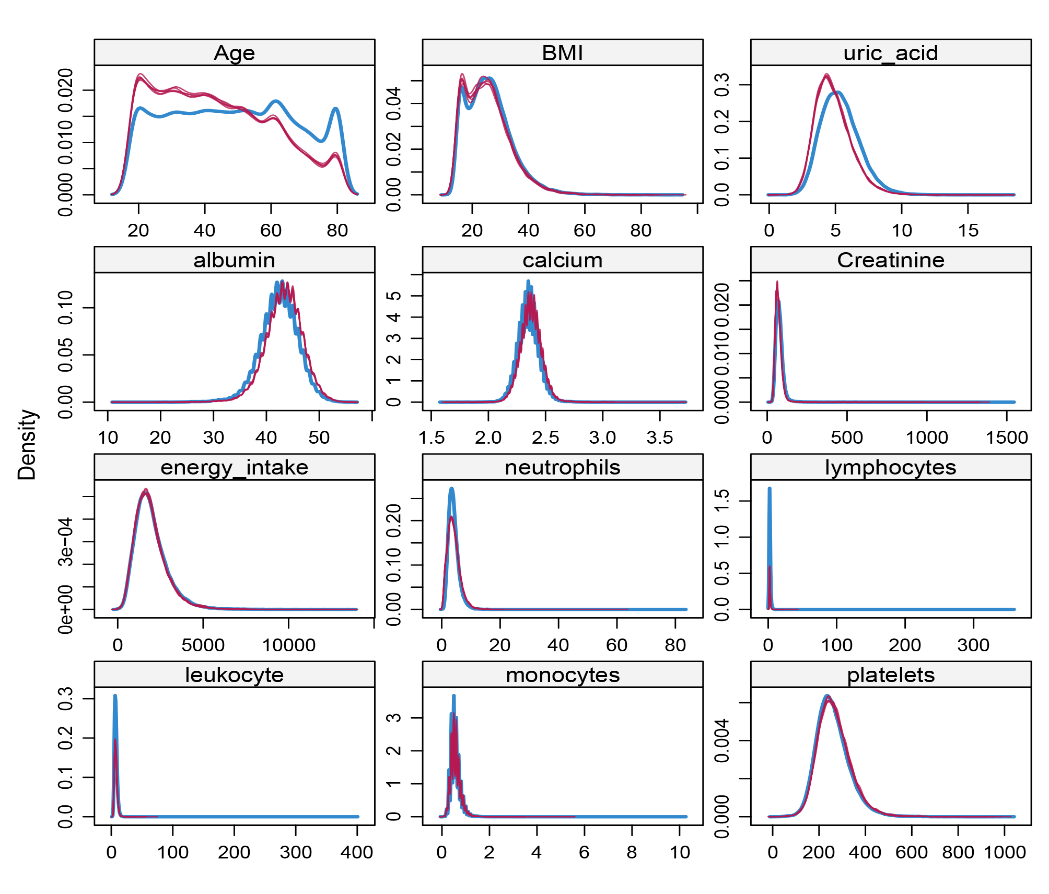
*

**Supplementary Figure 2. Density Distributions of Observed and Imputed Values**

*
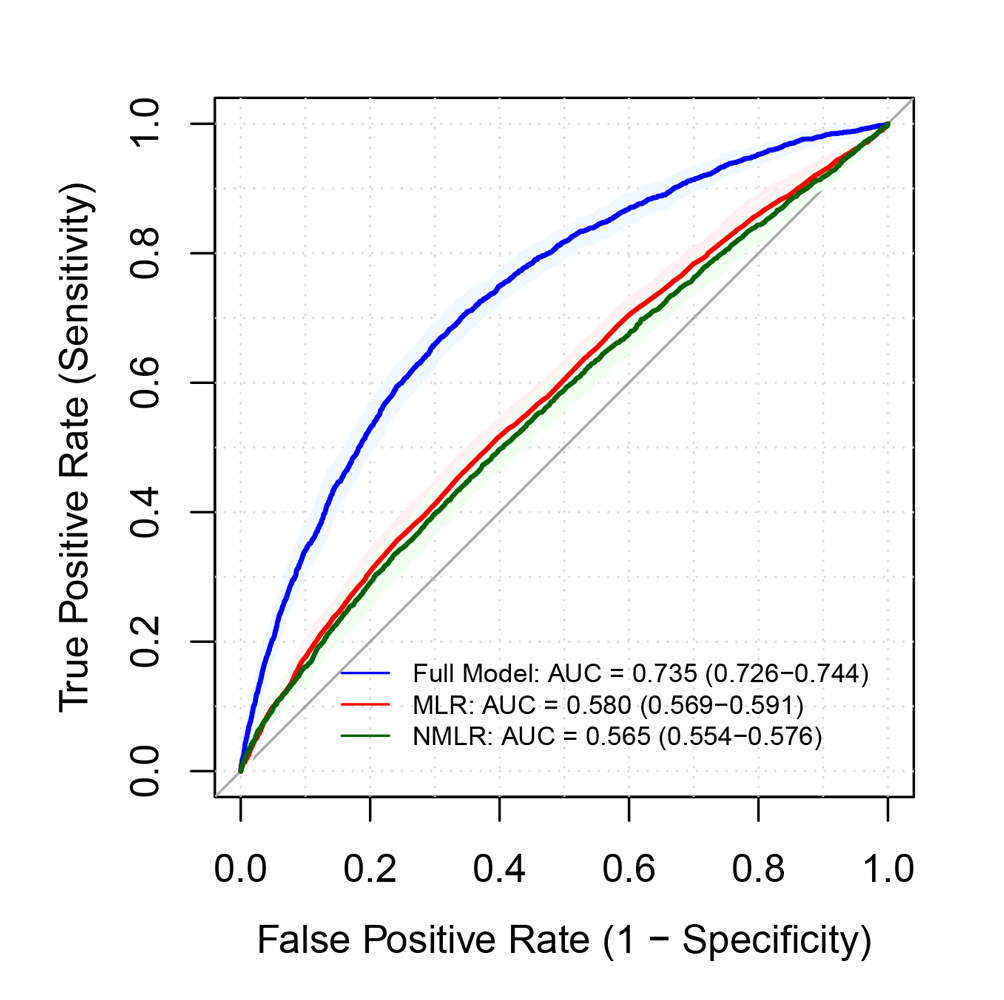
*

**Supplementary Figure 3. Bootstrap-Validated ROC Curves for the Final Prediction Model**
